# Exploring general practice staff perspectives on a teaching concept based on instruction videos for diabetic retinopathy screening – an interview study

**DOI:** 10.1080/02813432.2024.2396873

**Published:** 2024-09-03

**Authors:** Malene Krogh, Martin Bach Jensen, Morten Sig Ager Jensen, Malene Hentze Hansen, Marie Germund Nielsen, Henrik Vorum, Jette Kolding Kristensen

**Affiliations:** a Center for General Practice, Aalborg University, Aalborg, Denmark; b Department of Otorhinolaryngology, Head and Neck Surgery, Aalborg University Hospital, Aalborg, Denmark; c The Clinical Nursing Research Unit, Aalborg University Hospital, Aalborg, Denmark; d Department of Health Science and Technology, Aalborg University, Aalborg, Denmark; e Department of Ophthalmology, Aalborg University Hospital, Aalborg, Denmark

**Keywords:** Type 2 diabetes, diabetic retinopathy screening, artificial intelligence, general practice, video, teaching, qualitative research

## Abstract

**Objective:**

The aim of this study is to explore general practice staff perspectives regarding a teaching concept based on instructional videos for conducting DR screenings. Furthermore, this study aims to investigate the competencies acquired by the staff through this teaching concept.

**Design and setting:**

Qualitative cross-sectional study conducted in general practice clinics in the North Denmark Region.

**Method:**

A teaching concept was developed based on instruction videos to teach general practice staff to conduct diabetic retinopathy screenings with automated grading through artificial intelligence. Semi-structured interviews were performed with 16 staff members to investigate their perspectives on the concept and acquired competencies.

**Results:**

This study found no substantial resistance to the teaching concept from staff; however, participants’ satisfaction with the methods employed in the instruction session, the progression of learning curves, screening competencies, and their acceptance of a known knowledge gap during screenings varied slightly among the participants.

**Conclusion:**

This study showed that the teaching concept can be used to teach general practice staff to conduct diabetic retinopathy screenings. Staffs’ perspectives on the teaching concept and acquired competencies varied, and this study suggest few adjustments to the concept to accommodate staff’s preferences and establish more consistent competencies.

## Introduction

Change is the only constant element within healthcare [1]. Aging population, changing disease patterns, and new treatment options due to technological improvements require staff to adapt to new demands almost constantly [1,2].

Artificial Intelligence (AI) has been applied in recent technological improvements within healthcare [2], and it is predicted as a potential transformational force which could help adapt to the increasing demands in primary care [3,4]. With its ability to automate task and assist diagnostics, AI has already affected different healthcare fields [2,3]. Within diabetes care, there have been notable advancements in task automation and workflow due to developments in AI [5,6]. For instance, diabetic retinopathy (DR) screenings, which are essential for detecting diabetes-related changes in the retina that may lead to visual impairment, automation of image grading through AI is now available [7]. This technological advancement has enabled the automation of previously time-consuming specialized tasks, potentially allowing non-specialists to effectively analyse images [7]. This offers a relocation of work task among healthcare staff, which potentially could relieve workload at Danish ophthalmology clinics challenged by long waiting time [8]. Few studies have explored implementation on DR screenings with automated grading into general practices in the Netherlands, USA and Australia in order to facilitate patients’ participation in the screening [6,9,10]. Nurses and other practice staff have demonstrated their ability to handle the task, and the AI grading results have proved successful validation based on its sensitivity, specificity, positive and negative predictive values. However, the existing studies warrant further research to explore more aspects of the implementation process in general practice.

For a thorough understanding of DR screening implementation in general practice, knowledge about the teaching process of staff to carry out the screening is crucial. This could involve determining effective teaching methods for acquiring the screening skills, as well as gathering perspectives from the learners to assess the effectiveness of these methods. However, studies [6,9,10] do not specify teaching methods or any criteria for competence establishment. Currently, there are no recommendations on how to teach general practice staff to perform DR screening with automated grading in general practice.

The effectiveness of teaching videos has been praised as being superior in ensuring performance outcomes and satisfaction compared to traditional teaching methods used in nursing education [11–13]. Moreover, research has demonstrated that short videos have the potential to improve the acquisition of clinical skills [13]. Videos can serve as an introduction before initial performance, but also as a quickly review before completing a clinical procedure [13]. Videos provide the opportunity to watch procedures repeatedly, and some prefer this method for its flexibility, self-management, and repetition [11,12]. The utilization of videos in teaching contexts also presents notable advantages in terms of cost-effectiveness and time efficiency, making video highly suitable for many [12]. Videos integrate visual and auditory cues [12], facilitating learning across multiple styles, such as visual, auditory, and kinaesthetic [14]. These learning styles serve as channels to receive and absorb new information and experiences [14]. While some individuals may have a preferred learning style, others benefit from a multimodal approach [14,15].

Given the limited knowledge regarding how to teach general practice staff to perform DR screenings with automated grading and considering potential benefits of using video in the teaching process, more research in this field is needed. The aim of this study is therefore to explore general practice staff perspectives regarding a developed teaching concept based on instructional videos for conducting DR screenings with automated grading. Furthermore, this study aims to investigate the competencies acquired by the staff through this teaching concept.

## Method

We emphasize the development and procedure of the teaching concept in the method section, as no similar concept for DR screening for general practice staff has been described. This is followed by a detailed description of the interview method.

### Study design, participants and setting

Participants in this qualitative cross-sectional study included staff involved in diabetes treatment from ten general practice clinics in the North Denmark Region, Denmark. The goal was integration of the screening, not staff designation, and participant selection was therefore always made in collaboration with clinics. Whether one or more participants was included from a clinic depended on the usual organization in the clinic. There were no participant exclusion criteria.

Participating clinics had the DR screening device for 2-6 weeks to screen patients with type 2 diabetes (T2D). Holidays, vacations, or difficulties in recruiting patients could extend the period. The clinics were responsible for recruiting patients for the study and were advised to start screening patients within the first couple of days after a scheduled instruction session, thus, their acquired knowledge could be utilized while it still retained in memory. The clinics were compensated for the time spend participating in the study.

### Teaching concept based on instruction videos

The teaching concept was developed in collaboration with an expert group, comprising: two experts in DR screening and six professionals currently working in and/or conducting research in general practice settings. The collaboration entailed planning the concept and discussing teaching material and its quality. MK was responsible for developing all teaching material, with ongoing feedback. During the development, personal experiences operating the fundus camera and software also helped shape the material.

The concept included a one-hour instruction session, instruction videos and a brochure describing how to perform the DR screening and operating screening tasks. An instructor (MK or MH) was present during the instruction session to initiate the session and present the materials. The instructor’s role was not to demonstrate the screening or provide verbal explanations, but rather to facilitate the use of teaching materials during the session if needed.

The material was evaluated by the expert group making sure language and visual aspects were comprehendible and logical. A pilot test of the instruction session was completed with one T2D treating nurse from a general practice clinic where both instructors were present. The pilot session provided an opportunity to evaluate the material and allowed instructors to practice and establish an agreed flow for the session. It was determined that a single pilot test sufficed, given the results of the initial evaluation.

### Components of the teaching concept

#### Instruction videos

Two instructional videos were developed consisting of both screen videos conducted using the OBS Studio Software and videos recorded using a OnePlus8 mobile phone. A Danish voiceover was recorded with a microphone (Røde NT-USB - USB Microphone). Videos and voiceover were uploaded to the software program Videoeditor available on Windows 10, in which editing was carried out.

The first videos focus was how to perform the screening, informing the viewer about correct patient seating, how to take the picture, etc. The duration for the first video was 5.51 min. The second video explained typical problems that could occur during a screening and their solution, e.g. the eye image would not be uploaded to the AI analysis software. The second video had a duration of 5.54 min.

Videos were stored on an USB stick. A 15 pages instruction brochure summarizing video content in concise text and illustrations was developed to supplement the videos.

The development process of the instruction videos follows recommendations derived from a theory-based approach to creating educational videos [13].

#### Required tasks

Six screening tasks were developed for staff to fulfil during the instruction session, aiming to establish a shared competency level. Four tasks involved operating the camera, and these tasks were developed to enhance participant’s familiarity with the screening and improve memory [13,16]. For the fifth task, participants were asked by the instructor to consider which actions they would take if the camera failed to capture an image of the patient’s eyes, encouraging them to reflect on various potential solutions. For the final task, the instructor presented four images on printed paper, varying in quality (one with good quality), and participant was required to accurately identify them based on their quality. Solutions to all tasks were explained in the instruction video and brochure.

#### Fundus camera

**Image 1.**
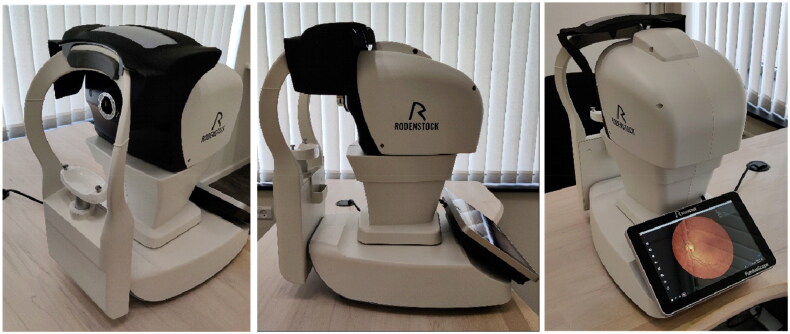
Fundus scope camera used for DR screening.

The camera used for fundus photography was a non-mydriatic camera (FundusScope, Rodenstock, Germany) that captures high-resolution images of the retina with a field of view of 45°. The camera has numerous automated features, ensuring user-friendly operation [17]. User interaction with the camera occurs *via* a connected tablet, where each patient’s screening data is logged using a unique ID number to link patients with their fundus image. Upon capturing an image, it is instantaneously transmitted to the tablet. The camera is illustrated on Image 1.

The camera was delivered personally and set up in the participating clinics. Optimally, the room for the screening procedure could be darkened, as lighter rooms may lead to small pupils and thereby poor image. Mydriatic eye drops were not used in the screenings.

#### Automated grading used to support staff in DR screening

When an image was captured by the fundus camera and transmitted to the camera tablet, data was subsequently sent to an AI analysis software for analysis (RetinaLyze System; RetinaLyze A/S, Hørsholm, Denmark). When images were analysed, results displayed as a colour code, green; indicating no DR changes, yellow; few changes, red; several changes. Image analysis was completed within 15 s. The AI analysis software has previously been described and tested [18].

#### Procedure of the teaching concept

The one-hour instruction session was initiated with a brief oral introduction made by the instructor. The participant watched the instruction videos on a portable computer and was afterwards able to operate the camera, re-watch videos, or read the brochure depending on their preference. The screening tasks were provided in printed form and had to be solved before the end of the session.

The instructor acted as patient during the session, however, when more participants took part in the session, they typically performed the screening on each other. The instructor would not interfere with the participant’s interaction with the camera, only observe the process. However, when questions arose, a dialog was initiated about video or brochure content.

With about 15-20 min left of the session, or when a couple of screenings successfully had been performed, the participant was informed to solve the screening tasks. For the two last tasks, the participant would verbally inform the instructor how they would approach solving the hypothetical problem and assess the image quality.

After the instruction session, the participant was encouraged to continue their learning process to maintain acquired competencies and enhance their experience before screening their first patient. How participants chose to maintain competencies was left to their preference, but examples were provided, such as practicing on colleagues, reviewing instruction videos or brochure, or revisiting screening tasks.

It was recommended to schedule first patient close to the instruction session, as competencies and knowledge might be easier to recall. This information was previously conveyed during clinic’s agreement to study participation.

## Semi-structured interviews

### Data collection

Semi-structured interviews were performed with the participants after the clinic had completed participation in the study [19]. This allowed participants to test their acquired competencies from the instruction session in a real-life setting. The interviews were performed in an undisturbed room in the participating clinics by MK or MH.

The aim of the interviews was to explore staffs’ perspectives on the teaching concept and their acquired competencies. The interviews were based on an interview guide developed by the author group, and the interview guide was adjusted between interviews by integrating inputs from previous interviews to the following [19]. The interviews were performed in Danish and were audio-recorded on a Sony ICD-PX370 Digital Dictaphone.

### Data analysis

The author MK conducted an inductive thematic analysis guided by the principles and guidelines of analysis [20]. All interviews were first transcribed verbatim in Microsoft Word and transferred to the software program, Nvivo 14, for further data analysis. During transcriptions initial notes were made. After transcriptions of all interviews, initial coding began with further familiarization of the data, and all transcriptions and notes were read through. Codes and sub-codes were made by looking for patterns and perspectives that appeared in the interviews with relation to the study aim. Themes and sub-themes were generated analysing the codes. The generation of both codes and themes involved an iterative process, with frequent revisions to the codes and transcriptions for refinements of the codes or themes. Quotes were selected with the purpose to demonstrate how the findings arose from the data. Throughout the analysis process, MK collaborated with a peer, MH, for sparring and discussion with the purpose of quality assuring the analysis steps. MH had familiarized herself with five interviews, and several meetings were held to discuss developed codes, themes, labelling of codes/themes, the overall context and reporting the findings.

### Ethics

The study obtained approval from the Committee on Medical Research Ethics, protocol number: 2200781. All clinics and participants provided informed consent prior to participation in the study and all study data were pseudo-anonymised using de-identification numbers. Only the principal investigators knew the identity of the participants.

## Results

The 16 general practice staff members taking part in the study are describes in Table 1 which includes information on participant number, gender, age, and job title. The table show that the vast majority were female nurses, but two female participants had job titles as biomedical laboratory scientists or social and healthcare assistants. Only one male participated, who also was the only general practitioner (GP). The average age of the participants was 51 ± 6 years. One participant stopped the screening procedure ahead of time but participated in the interviews. The average interview duration was 37 min, ranging from 22 to 60 min.

**Table 1.** Characteristics of participants.

Participant NO	Gender	Age	Job title
P1	Female	56	Nurse
P2	Female	49	Nurse
P3	Female	61	Nurse
P4	Female	65	Nurse
P5	Female	37	Nurse
P6	Female	52	Nurse
P7	Female	55	Nurse
P8	Female	51	Nurse
P9	Female	44	Nurse
P10	Female	51	Nurse
P11	Female	44	Nurse
P12	Female	46	Nurse
P13	Female	52	Nurse
P14	Female	49	Biomedical laboratory scientist
P15	Female	47	Social and health assistant
P16	Male	59	General practitioner

### Themes

Four main themes were generated from the interviews with associated sub-themes, as presented in Table 2.

**Table 2.** Themes and sub-themes.

Themes	Sub-themes
Preferred learning style	Instructional videos
	Absence of physical demonstration
	Favor kinaesthetic learning
	Peer-learning and collaboration
Individual learning curves	Perceived difficulty
	Initial uncertainties
	Readiness after instruction session
	Screening memory affected by interval
Acquired screening competencies	Image assessment
	Problem solving strategies
Knowledge gap	Usual DR screening analysis procedure
	Satisfaction level with gained knowledge
	Unable to answer questions
	Professional knowledge has its limits

### Preferred learning style

A key finding of this study was that the participant expressed preferred learning styles which influenced their perception of the methods employed during the instruction session.

Using instruction videos and brochure was generally accepted; however, two participants wanted more information about technical overview and one participant wanted more images with examples of good and poor-quality images in the material. The length of the videos was considered appropriate, but two participants mentioned difficulty in retaining video content and expressed the need to wait until operating the camera themselves. One stated:

Well, you know, I’m the type who can’t really remember stuff until I’ve got it in my hands. You gotta have it in your hands and try it out a few times. And now, as I’m getting older, I now realize that. It’s not something that stresses me out like it did 20 years ago. (P2)

Kinaesthetic learning, i.e. being physically active in the learning process, was favoured and highlighted as fundamental in learning situations by all participants, as it is where genuine learning takes place, which one participant stated as:

I actually think the session was sufficient. We also did some practical tasks, and that’s probably where you really learn it – you know, it’s likely during those hands-on activities. (P16)

Four participants mentioned they would favour a physical demonstration of the screening process during the instruction session, and when asked whether the session could work without personal initiation by an instructor, it was suggested to rely on the individual’s preferences and technical skills.

Some participants preferred peer learning facilitated by colleagues and mentioned that collaboration is common in daily work routines. This was supported by the fact that some participants with longer intervals between the instruction session and first patient screening chose to receive instruction from colleagues rather than attempting the screening themselves with guidance from the teaching material. For example:

So, it was a good thing that someone had been here while I was off for a few days. Someone who could kinda say, like, ‘Then you press that button, and then…’. So, you know, maybe I could have pulled up the video again, I don’t know, but that’s not what I did. (P7)

### Individual learning curves

Individual learning curves appeared among the participants, with some learning at a faster pace than others. This was reflected in participants feeling of readiness to conduct screenings after completing the instruction session, and many continued practicing on each other or other colleagues after the session to improve competencies and readiness.

The participants describe competence progression over time and routine establishment, and one participant stated the repetition of new tasks as important:

I mean, every time you need to learn something new, no matter what it is in life, you just have to give it a try a few times before it clicks. (P3)

A sense of uncertainty was associated with initial screenings on patients. Participants expressed this through extra preparations, such as double-checking camera functionality before screenings or conducting initial patient screenings in pairs. Four participants ended up with a relative long interval (>9 days) between the instruction session and first patient screening, and it was clear that the interval influenced the recall of screening procedure, and one participant acknowledge that shorter intervals would be ideal, but not always a possibility:

Well, it took a bit of time before we got started. And I was actually thinking about that – I had actually forgotten it, so it [the screenings] has to be planned pretty quick if you need to remember it. But, of course, you also need to get a hold of the patients, have the time, and it must fit into the schedule. (P10)

All participants describe the screening as intuitive and easy to operate, except one participant, who described a lack a technical overview of the screening from the start:

So, in the beginning, I was really unsure about what it was, like, I had a hard time to grasp the overview – I mean, what kind of programs they were, what they did, and what I was doing. (P4)

The lack of technical overview hindered successful execution of screenings, and the participant chose to withdraw from the study ahead of time due to persistent technical issues after attempting to screen three patients.

### Acquired screening competencies

A theme that became clear through the analysis was how participants’ competencies were reflected in their actions during screening and problem solving. Image quality assessment varied from person to person, and most took image quality into account when performing the screening:

I actually thought it was really easy to work with, and you could quickly tell if the images were good enough. I mean, you got a completely clear picture of the eye and the optic nerve, along with a lot of tiny blood vessels. (P14)

Two participants mentioned they did not assess image quality, and one referred to trusting the machine to point out poor quality images:

I thought the machine would tell me if it wasn’t satisfied with the image. (P7)

The participants ability to solve problems effectively was quite noticeable. Most participants had many ideas on how to solve issues, leading to the exploration of different solutions when issues occurred. One example was when a problem with small pupil size arose:

I asked him [a patient] to move his head away from the chinrest, and then I asked him to close his eyes for a bit and rest with his eyes closed. Then I said, ‘In a little while, I’ll tell you to open your eyes, and then I’ll take the picture’. (P9)

When problems occurred that participants could not solve from intuition, participants’ actions differed. Some chose to consult the instruction brochure for guidance, while most preferred seeking assistance from colleagues, where some looked in the brochure together. Having more people skilled to perform the screening in a clinic was generally appreciated, one participant expressed:

It was a good thing that we were two, so I could just go get her [colleague] if something was not working, or ask, ‘How was it with this again?’ (P13)

### Knowledge gap

It was clear that a knowledge gap existed involving a lack of fundamental understanding on usual DR screening procedure and analysis. Staff’s perceptions of this knowledge gap varied widely. Most were satisfied with the knowledge they had acquired, feeling they had learned to perform the screening sufficiently:

The only expectation I had was to learn to use the device, and I think we learned that quite well. (P1)

The absence of more background knowledge was especially unsatisfactory for one participant, fostering a sense of being dumb in the screening situation:

I need to be more knowledgeable than the patient, so I can answer questions. Because some of the patients, who regularly visit the ophthalmologist, when they came into the room with me, they actually knew more than I did, because I don’t have diabetes, I don’t go to the ophthalmologist… so there I am, actually feeling a bit dumb in the situation. (P8)

The knowledge gap also became clear in participants’ inability to answer in-depth questions from patients. Three participants expressed concern regarding the knowledge gap, hoping to avoid further questions during consultations. However, most participants acknowledged that limitations in professional knowledge are normal, and this is also observed in other daily scenarios where patients are referred to GPs or specialists for further follow-up:

I think, especially as nurses, we can easily say to patients, ‘I simply don’t have enough knowledge about how this affects you, so that’s why you need to see an ophthalmologist’. We also use a similar approach with many other things in our daily work… Patients are usually understanding of this, as they know we occasionally put up a stop sign. (P12)

Similarly, in this context, there was a consensus from participants that ophthalmologists must deal with additional questions from patients.

## Discussion

### Principal findings in relation to other research

This study found no substantial resistance to this teaching concept from the participants; however, participants’ satisfaction with the methods employed in the instruction session, the progression of their learning curves, their screening competencies, and their acceptance of a knowledge gap during screenings varied slightly.

The extent to which individuals appreciated the teaching methods employed during the instruction session varied, particularly concerning the utilization of videos. Video based learning has previously been emphasized as a favourable teaching method compared to traditional methods [11–13], however it is impossible to meet everyone’s preferences through video [12]. Where other learners has criticized video length or preferring to read information rather than viewing [12,21], we did not find video length an issue, but some participants preferred physical demonstration instead of viewing videos. In relation to learning styles [14], the developed teaching concept includes multiple styles to a greater or lesser extent, and we hypothesize that if participants’ preferences have not been met using instructional videos, their preferred learning style likely becomes relevant at a later stage during the teaching concept.

Participants used their peers during the learning phase, and some preferred getting help from colleagues rather than attempting to finds answers themselves in the teaching material. Valuation of peers is usual when learning new clinical skills. A systematic review and meta-analysis in medical education has shown that peer learning offers significant advantages in acquiring new skills [22]. Peer learning is especially appreciated when students enter their clinical stages of education, where more time often is spend on acquiring skills in clinical settings than on didactic teaching sessions [22]. Peer learning is also particularly well-suited for the instruction of practical and procedural clinical skills, potentially leading to enhanced practical assessment outcomes [22]. In our study, we did not test the effectiveness of peer learning and can therefore not clarify potential advantages it may have provided. Nevertheless, when considering the feedback from participants who had colleagues also capable to perform the screening, colleagues were highly appreciated, and all participants described using each other to a greater or lesser extend throughout the process.

It is natural for individuals to acquire competencies at different rates [23], and this phenomenon was also observed in this study. We found that acquired competencies after the instruction session influenced some participants’ feeling of readiness to conduct DR screening. Generally, more uncertainty was described during the initial screenings, but as experience grew, a routine establishment occurred. Learning new skills can be described in multiple stages, and as learner’s competence in performing specific skills improves, cognitive demands decrease, and the task becomes increasingly automated for the individual [24]. When participants in this study describe routine establishment, it may indicate a shift towards the automation of the screening process, whereas the initial screenings demanded more cognitive effort.

A qualitative study concerning learning strategies for GPs to attain point-of-care ultrasound competence state the importance of continuous training for sustaining acquired competencies [25]. Our study supports this statement, and it is essential to preserve the acquired competencies through regular screening activities, as longer intervals between screenings encountered challenges in recalling the screening procedure.

Performing DR screening with automated grading enable non-experts to perform the task [7]. Thus, it might therefore not be necessary for the screener to possess extensive knowledge on DR and grading process. Banerjee et al. [26] investigated the impact of AI on clinical education and found that one barrier associated with implementing AI is the potential reduction in clinical judgment and decision-making, as well as healthcare staff losing their accountability [25]. Our study revealed that three participants were concerned about their perceived lack of knowledge throughout DR screenings, leading to nervousness about handling questions from patients. This may align with the findings of Banerjee et al. [26], suggesting that these participants may have perceived their clinical judgment and accountability as insufficient in screening situations compared to their competence in other clinical procedures. Most participants were satisfied with their acquired knowledge, and it was mentioned as normal to refer to specialists for more in-depth answers in daily practice. It is our belief that when general practice staff use AI for automated grading in this screening, the premise is that the screener does not possess the same level of knowledge as an expert in DR screening. If that where the case, one of AI’s benefits, task relocation, might not be as advantageable if expert knowledge still was expected. However, we do still acknowledge that a few participants expressed a concern about their established knowledge to perform the screening.

### Strengths and weaknesses of the study

To our knowledge, this is the first study to provide a clear description on how general practice staff can be taught to conduct DR screening with automated grading. We recognize the potential for adjustments, however our study offers valuable insights that can serve as a foundation for further development and refinements of the teaching concept. A clear strength of our study is the insight of staff’s perceptions of the teaching concept and acquired competencies.

The learning theorist David A. Kolb argues that learning occurs through experiences and emphasizes the importance of learning in a real-life setting [27,28]. Creating a learning environment in the clinical setting, as this teaching concept does, is therefore a strength of the study and the developed concept.

In Denmark, the allocation of responsibilities in general practice clinics varies regarding patients with T2D. It was standard practice in almost all participating clinics (as well as in numerous other Danish general practice clinics) to delegate consultations and tasks pertaining diabetes patients to nurses, under the supervision of GPs, resulting in most participants in the study being nurses. In contrast, in other clinics, it may be the GP or other staff who manage consultations with diabetes patients. A limitation of our study in terms of generalizability is therefore the participation of only one GP and a very homogeneous composition among the study participants. This limitation should also be considered when assessing the feasibility of the teaching concept in clinics which have similar or different workflow from the participating clinics. Additionally, the healthcare systems and allocation of responsibilities in other countries may differ, which could impact the applicability of our findings internationally.

This study does not include data on long-term retention of learning, which would have enhanced our understanding of the durability of knowledge gained from the concept. Additionally, there is no quantitative measure to evaluate staff’s competency development over time or a specific competency target. Although we attempted to address this limitation using required screening tasks during the instruction session, it became evident that participants’ acquired screening competencies varied.

Through our findings, it becomes evident that a degree of confusion existed among a few participants regarding the assessment of image quality, highlighting a limitation of the study. It is worth noting that image quality assessment was addressed in instruction videos and text, and participants were required to assess image quality in one of the six tasks during the instruction session. Nonetheless, it might have been beneficial to provide a clearer description for study participants on whether image quality assessments were required during screenings.

## Implications for practice and research

The workload in general practices is high and practice staff often have busy schedules [29], and one advantage of this teaching concept is its flexibility and applicability in the clinical setting. Although the concept’s effectiveness without verbal initiation by an instructor remains uncertain, resource efficiency and cost-effectiveness, both with and without an instructor, might potentially be lower compared to traditional methods such as physical courses or lectures [30]. In addition to the economic benefits, completing the teaching concept without an instructor makes the implementation of the concept in general practices fast and empowers individual clinics to determine when they have time for completing the teaching. However, removal of the instructor from the teaching concept calls for further investigation to establish the potential disadvantages and advantages it may cause.

Besides testing the teaching concept without instructor initiation, we recognize the potential for its further development. Building on our findings, we offer the following recommendations:

*Include more information about DR in teaching material.* We recommend that the teaching material includes more information about DR. This involves what DR is and how it progresses, as well as more information on the DR screening procedure conducted by ophthalmologists. We believe that it is unnecessary for staff to be able to identify DR changes or perform manual image analysis, as this is the foundation of using AI analysis.
*Facilitate peer learning and collaboration.* Many participants mentioned peer learning and collaboration as beneficial when learning and during problem-solving. We therefore believe it is favourable for multiple staff to be able to perform the screening in the clinic.
*Develop clear national/international guidelines on acquired screening competencies.* We suggest a development of clear national/international guidelines on acquired screening competencies for general practice staff to conduct DR screenings. It is fundamental that DR screenings with automated grading are performed correctly to avoid poor image quality, which ultimately affects the validity of the results. However, there is a lack of evidence-based guidelines regarding the required competence level for general practice staff to perform the screening. Developing such guidelines may help ensure common competency goals are met in the future.

These recommendations can guide future research and development efforts to enhance the teaching concept for DR screening with automated grading in general practice.
